# “Pain talk”: A triadic collaboration in which nurses promote opportunities for engaging children and their parents about managing children’s pain

**DOI:** 10.1002/pne2.12061

**Published:** 2021-08-09

**Authors:** Abbie Jordan, Bernie Carter, Konstantina Vasileiou

**Affiliations:** ^1^ Centre for Pain Research and Department of Psychology University of Bath Bath UK; ^2^ Faculty of Health, Social Care and Medicine Edge Hill University Ormskirk UK; ^3^ Department of Psychology University of Bath Bath UK; ^4^ University of West Attica Athens Greece

**Keywords:** children, communication, nurses, pain, pain assessment, pain management, parents

## Abstract

Effective communication with children about pain is important and has the potential to mediate the short‐ and longer‐term effects of pain on children. Most communication studies relating to children's pain have focused on language children use to describe everyday pain experiences. However, little is known regarding how health professionals, particularly nurses, communicate with children in healthcare settings about pain. This study aimed to explore how nurses talk to children and their parents about pain and what factors influence nurses’ use of language and non‐verbal communication. A cross‐sectional mixed‐methods (predominantly qualitative) survey (“pain talk”) was conducted, comprising qualitative items about pain communication and four vignettes portraying hypothetical cases of children representing typical child pain scenarios. Participants were recruited via email, social media, newsletters, established networks, and personal contacts. A total of 141 registered (68.1%) or in‐training nurses across 11 countries with experience of managing children's pain completed the survey. Textual survey responses were analyzed using conventional qualitative content analysis. Qualitative content analysis generated a meta‐theme “Being confident and knowing how to do ‘pain talk’” and four main themes that described the functions, purpose, and delivery of “pain talk”: (a) “contextualizing and assessing,” (b) “empowering, explaining, and educating,” (c) “supporting, affirming, and confirming,” and (d) “protecting, distracting, and restoring.” “Pain talk” was a triadic collaborative communication process that required nurses to feel confident about their role and skills. This process involved nurses talking to children and parents about pain and creating engagement opportunities for children and parents. “Pain talk” aimed to promote the agency of the child and parent and their engagement in discussions and decision‐making, using information, support, and comfort. Nurses shaped their “pain talk” to the specific context of the child's pain, previous experiences, and current concerns to minimize potential distress and adverse effects and to promote optimal pain management.

## Graphical abstract

Analyses of international qualitative survey data identified that nurses engage with ‘pain‐talk’ when talking to children and parents about child pain. This ‘pain‐talk’ focuses on emphasising the agency of the child and parent, taking account of the child's specific context and considering previous pain experiences to maximise effective pain management.

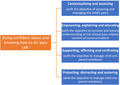

## 1 INTRODUCTION

Pain is a common experience in childhood and is typically experienced from a very early age,^1^ continuing throughout childhood. A recent study highlighted the common nature of pain in toddlerhood, reporting that children aged 1‐3 years experienced an average of 1.02 pain incidents per child for each hour spent in a soft play setting.^2^ In addition to everyday bumps and scrapes, children's experiences of pain extend through experience and discussion around painful procedures such as venepuncture and vaccinations.^1^ For many, childhood pain experiences comprise acute pain incidents such as those described above, but for a minority of children and young people, instances of acute pain continue, resulting in the experience of chronic pain.^3^ Some children and young people experience chronic pain as a condition^4^ in itself while others experience chronic pain as part of managing a long‐term pain‐related condition such as Crohn's disease.^5^ For these children and young people, pain may also be experienced as a result of acutely painful condition‐specific procedures such as injecting methotrexate, with such regular acutely painful procedures being distressing for children and young people on children's quality of life.^6^

It is widely acknowledged that pain in children is both under‐reported and under‐treated, even in hospital settings.^7^, ^8^ Appropriate management of pediatric pain is critical for multiple reasons including improving child well‐being and reducing child distress in real time. Recent work has highlighted the potential longer‐term effects of poor pain management in children, with evidence showing that children's memories around management of their pain can influence their experience of subsequent pain.^9^ Such work highlights the critical importance of “good” communication about pain in pediatric clinical settings.

Existing studies concerning communication of pediatric pain have examined the language that children use to describe everyday pain^10^, ^11^, ^12^ across developmental trajectories. Such work has typically focused on the experience of everyday pain within a home setting. However, conversations also frequently occur in healthcare settings when children are undergoing painful procedures such as venepuncture or presenting in healthcare settings with pain associated with long‐term physical health conditions such as juvenile idiopathic arthritis.^13^, ^14^ Consequently, it is important to address how healthcare professionals communicate with children (and parents) about pain regarding both language and non‐verbal behavior. While such pain‐related conversations occur with a range of professionals in healthcare settings, it is nurses who undertake the greatest number of pain‐related interactions with children with regard to assessing and managing pain.^15^ Consequently, this study focused on exploring the experiences of nurses concerning communicating with children and parents about pediatric pain.

This paper reports on analyses of nurses’ responses to pediatric pain vignettes and questions about their confidence which formed Phase 1 of a mixed‐methods study to explore nurses’ “pain talk” with regard to managing children's pain. Findings from Phase 2 (qualitative interviews with a smaller sample of nurses) focus on the role of reassurance in “pain talk”.^16^

## 2 METHODS

This study aimed to explore how nurses working with children talk to children and their parents about pain and what factors influence nurses' language and non‐verbal behaviors.

### 2.1 Design

A cross‐sectional, mixed‐methods (predominantly qualitative) survey was used. The survey included four distinct vignettes which portrayed scenarios in which a child or young person was experiencing pain. The survey was designed by two of the authors, both female (AJ, psychologist and BC, children's nurse) and both of whom have substantial experience of working in the area of pediatric pain.

There are numerous benefits of surveys that are either wholly or predominantly qualitative in nature. Qualitative surveys are resource‐lite and offer the opportunity to explore a broad range of views which can be more focused on the topic of interest.^17^ As part of the survey, we also employed the technique of vignettes to further elicit participants' views and practices “grounded” in specific situations.^18^

### 2.2 Participants

This study involved recruiting a large international sample of nurses (n = 141) with experience of managing children's pain. Participants were recruited to complete an online survey about how nurses talk to children and parents about pediatric pain. Eligible participants were required to be aged 18 years or over and be either (a) a registered nurse or equivalent whose work focused on children and parents/carers or (b) training to be a registered nurse or equivalent whose work (clinical/managerial/academic, etc) focused on children and their parents/carers.

A total of 141 nurses from eleven countries (UK, Ireland, Canada, USA, Australia, Iceland, Sweden, Qatar, Turkey, Switzerland, and Jordan) participated in this study. Of those nurses, 68.1% held a children's nursing qualification; 95.0% identified as women and 92.9% were White (Table 1). Most of the participants (63.1%) were residents in the UK, and most (68.8%) had trained in the UK. The sample represented preregistration students undertaking a course to gain a children's nursing qualification (27.7%), through to nurses who had been qualified for <5 years (32.6%) and nurses who had been qualified for >20 years (38.3%). Of the qualified nurses (n = 101), 76.2% were working in clinical settings, 12.8% in academic settings, 6.9% in other settings (eg, school), and 3.9% were studying for a course.

**TABLE 1:** Participant demographic and work‐related characteristics

Gender	
Women	134 (95%)
Men	6 (4.3%)
Prefer not to say	1 (0.7%)
Ethnicity	
White	131 (92.9%)
Mixed heritage	4 (2.8%)
Asian	3 (2.1%)
Missing values	3 (2.1)
Country of residence	
UK	89 (63.1%)
Canada	20 (14.2%)
Ireland	9 (6.4%)
Australia	9 (6.4%)
USA	2 (1.4%)
Iceland	1 (0.7%)
Sweden	1 (0.7%)
Jordan	1 (0.7%)
Qatar	1 (0.7%)
Turkey	1 (0.7%)
Switzerland	1 (0.7%)
Missing values	6 (0.7%)
Place of nursing training	
UK	97 (68.8%)
Europe (other than UK)	12 (8.5%)
North America	21 (14.9%)
Australasia	9 (6.4%)
Asia	2 (1.4%)
Years since first qualified as a nurse	
<5 years	46 (32.6%)
5‐10	15 (10.6%)
11‐15	8 (5.7%)
16‐20	12 (8.5%)
More than 20 years	54 (38.3%)
Missing values	6 (4.3%)
Qualification status and setting	
Qualified nurse currently working in a clinical capacity	77 (54.6%)
Qualified nurse currently working in academia	13 (9.2%)
Qualified nurse currently working in another setting (eg, school)	7 (5.0%)
Qualified nurse currently undertaking a course to gain a children's nursing qualification	4 (2.8%)
Preregistration student nurse currently undertaking a course to gain a children's nursing qualification	39 (27.7%)
Missing values	1 (0.7%)
Children's nursing qualification	
Yes	96 (68.1%)
No	44 (31.2%)
Missing values	1 (0.7%)
Particular work setting currently working in	
Specialist children's hospital	36 (25.5%)
Children's setting in a general hospital	37 (26.2%)
Community setting	20 (14.2%)
University / other education settings	45 (31.9%)
Missing values	3 (2.1%)

### 2.3 Procedure

The study received ethics approval from the Psychology Research Ethics Committee at the University of Bath and Edge Hill University.

A snowball sampling approach was adopted, with the target population reached via invitations being posted via email, social media (eg, Twitter), Web sites, newsletters, via established networks, personal contacts and key nursing, and healthcare organizations in the UK and internationally (eg, Royal College of Nursing Children and Young People's Forum, British Pain Society). The invitations contained a link to the front page of the survey which provided information about the study and consent statements. Once the participant had read the information and endorsed the relevant consent statements, they could access the survey. All participants had the right to withdraw up to the point of submission of the survey.

The survey was administered online using Qualtrics software. The survey comprised three domains of questions. The first domain used both close‐ and open‐ended questions to collect demographic (ie, gender, ethnicity, country of residence) and work‐related information (ie, job title, country of undertaking nursing training, years since first qualified as a nurse, qualification status, whether they held a children's nursing qualification, current work setting, age groups of children they were working with and experience of training in managing pediatric pain). The second domain included general questions (n = 9) relating to pain communication with children and parents (five open and four closed questions). This section included four closed questions about their level of confidence (1 = not at all confident, 5 = very confident) in talking to a child and parent about pain, and their confidence that the child and the parent understood what they had said about managing pain. These four quantitative items were included to contextualize the qualitative data. The last section of the survey presented the vignettes. These vignettes presented four hypothetical cases of children experiencing pain and were developed by the researchers to represent different pain scenarios according to type of pain (eg, acute pain, chronic pain), child gender, and age. Parents were mentioned in two of the vignettes (Josh and Sati) in relation merely to being present with the child in the clinical setting. Participants were asked to complete six open‐ended questions around pain communication in relation to each vignette. The order of the vignettes was randomized, and participants were invited to answer the questions of as many vignettes as they wished. Participant data were retained if they had answered the questions linked to at least one vignette. All four vignettes are presented in Table 2. Participants were then asked to respond to specific questions about each of the vignettes concerning: (a) What they would say to the child and parents; (b) how they would approach the situation; (c) whether anything would enhance their communication in that situation; (d) their perceived confidence in managing that situation; and (e) how often they have managed a child in this particular situation previously. Appendix S1 presents the full details of the survey questions, including the vignettes.

**TABLE 2:** Details of the vignettes

Vignette name	Vignette detail
Josh	Josh is 9 years old and was admitted to hospital via A&E having developed “really bad pain” in his tummy at school. You meet Josh and his mum on the surgical ward.
Sati	Sati is 3 years old and is attending the hospital with her Dad. She is about to have a dressing on her arm changed. This procedure could be painful.
Mikel	Mikel is 4 years old and you want to give him some ibuprofen for his pain following a tonsillectomy.
Lisa	Lisa is 10 years old and you want to give her some paracetamol for her pain. She has a fractured wrist which has just been put into a splint.

### 2.4 Data analysis

The responses to the open‐ended questions were analyzed using the conventional approach to qualitative content analysis.^19^ This variant of qualitative content analysis constitutes an inductive analytic approach whereby the codes are generated directly from the data. Conventional content analysis is an appropriate analytic choice when the aim of the research is to describe the phenomenon of interest while staying grounded in the actual data.^19^ Our analysis proceeded in three stages to ensure that we maintained an iterative process between developing codes, assigning data to codes, and refining the developing codes while managing a voluminous corpus of data. Initially, we coded the data of the first 40 participants, creating the codes inductively. At this stage, we reviewed the codes and data, refining the coding scheme as necessary. Subsequently, we coded data of the next 40 participants, also further refining the coding scheme where required considering the additional data (eg, creating new codes and merging codes). Using the latest version of the coding scheme, the data of the final 61 participants were coded. Initial coding was conducted by KV (female, psychologist) and was discussed, revised, and defined in regular meetings with AJ and BC. After completion of coding, the researchers developed a preliminary analysis that grouped related codes into broader thematic domains. These iterative discursive processes were critical in terms of ensuring that analyses were credible and also grounded in the data, demonstrating analytical quality.^20^, ^21^ This preliminary analysis was further revised, with the final results being presented in the next section. The analysis of the four closed questions relating to confidence levels was undertaken using descriptive statistics (mean confidence levels and standard deviation) (see Appendix S2).

## 3 RESULTS

A total of 63 (44.7%) participants responded to pain‐related questions concerning solely 1 vignette, 35 (24.8%) participants to only 2 vignettes, 11 (7.8%) participants to only 3 vignettes, and 32 (22.7%) participants to all 4 vignettes. On average, participants replied only to the questions of 2 patient vignettes (Mean = 2.08, SD = 1.19). With regard to completion of specific vignettes: n = 76 (53.9%) participants completed vignette 1 (Josh); n = 73 (51.8%) completed vignette 2 (Sati); n = 67 (47.5%) completed vignette 3 (Mikel); and n = 73 (51.8%) completed vignette 4 (Lisa). The analyses of participants' engagement and understanding of “pain talk” in their response to the vignettes which they completed are presented in the following section in addition to quantitative findings pertaining to participants’ confidence ratings regarding engaging with and delivering “pain talk.”

### 3.1 The complexity of “pain talk”

Findings indicated that “pain talk” can be defined as a triadic collaborative process of communication, requiring confidence and involving the participants both talking and attending to children and parents about their pain and creating opportunities for the children and parents to engage in a reciprocal way. An important aspect of this “pain talk” was creating the optimal situation for the child to enable pain management and facilitating a space to create positive future memories of the pain‐related clinical encounter where possible. The focus was very much on use of “pain talk” as a collaborative communicative tool among nurses, children, and parents.

**FIGURE 1:**
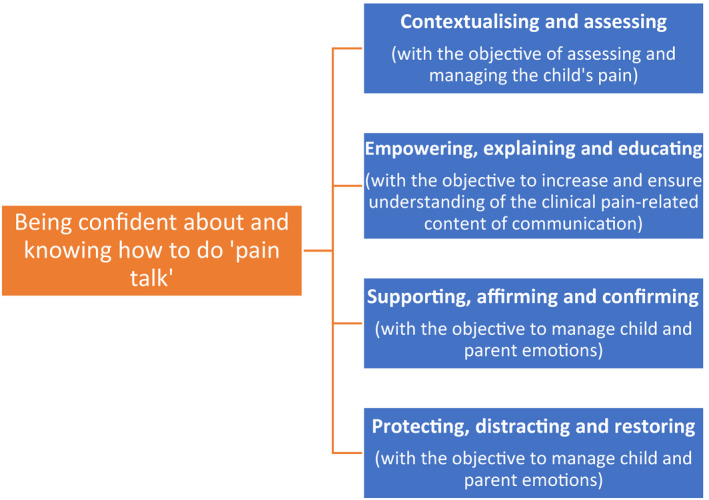
Relationship between the meta‐theme, the main “pain talk” themes, and the objectives of “pain talk”

Participants’ (nurses) “pain talk” with children and parents aimed to achieve three entwined objectives: (a) to assess and manage the child's pain, (b) to increase and ensure understanding of the clinical pain‐related content of communication, and (c) to manage child and parent emotions (see Figure 1).

Findings identified that nurses’ perceptions concerning their capability to deliver “pain talk” and knowledge around “pain talk” were dominated by a meta‐theme of “being confident and knowing how to do “pain talk.’” Within this meta‐theme, four main themes to describe the particular functions, purpose, and delivery of “pain talk” were generated. These themes comprised the following: (a) “contextualizing and assessing,” (b) “empowering, explaining, and educating,” (c) “supporting, affirming, and confirming,” and (d) ‘protecting, distracting, and restoring.” Each of these four themes will be explored in detail, with anonymized extracts from participants’ responses to the vignettes and open‐ended qualitative questions used as exemplars of the analytical points raised. Figure 1 provides an overview of the relationship between the meta‐theme, the four main “pain talk” themes, and the objectives of “pain talk.”

Effective “pain talk” was perceived as a tool to facilitate participants in understanding and assessing the child's pain condition, subsequently enabling the participant to recommend the appropriate pain management plan. “Pain talk” comprised a range of content, including participants asking specific questions about the nature and origin of the child's pain and using age‐appropriate pain scales as a means of both assessing pain objectively and improving their communication with children. Additional aspects of “pain talk” content included participants asking specific history‐related questions around analgesia (eg, what has previously been administered and how effective it had been). “Pain talk” also included decision‐making about treatment and explaining to children and their parents how the intervention would help the pain.

To achieve its aims of effective communication about pain between participants, children, and parents, “pain talk” was characterized by creating space for the child's agency, honesty, sensitivity, being open and truthful yet gentle and delicate, showing care, attention, empathy, friendliness, parity, listening actively and carefully, respecting their pace, and not rushing them. This talk was supported through non‐verbal communicative acts by participants including smiling, getting down to the level of the child, using a soft voice, eye‐contact, and gentle movements.

### 3.2 Meta theme: Being confident about and knowing how to do “pain talk”

A meta‐theme relating to nurses' perceived confidence to enact “pain talk” and their knowledge concerning “pain talk” dominated nurses' perceptions and experience around communicating to children and parents about pain. Participants’ ease with delivering “pain talk” and the content of their “pain talk” was influenced by organizational factors such as the lack of time available in clinical practice to dedicate to focused and effective communication:

“Time is usually the factor that inhibits good communication, [as] when not busy most patients & parents can be comforted, informed, guided, etc” (P57)

“Pain talk” was also influenced by self‐referential elements relating to participants' professional identity including factors such as their clinical knowledge, pain‐related work experience, and life skills. Participants' sense of competence in managing pain care procedures influenced their confidence in talking with children and parents about pain in addition to the ways in which they talked about pain. Experience was gained in different ways including length of service such as *“working in paediatrics for over 20 years” (P60)* and experience of managing of patients in certain clinical situations. Knowing how to do “pain talk” and having the diverse knowledge to underpin participants' work was considered critical. “Pain talk” knowledge encompassed specific clinical knowledge, knowledge of child development, and *“knowledge of appropriate interventions [that] relieve pain” (P41)*. Closely linked to knowing what to say were the life experiences (eg, being a parent) and skills (eg, observation) that facilitated participants to communicate that knowledge effectively. “Pain talk” was reported as being more successful and less challenging when participants perceived themselves as being experienced, knowledgeable, and competent. These qualitative findings pertaining to the relationship between confidence and the ability to enact effective “pain talk” are supported by our quantitative findings (see Appendix S2 for further details) which showed that the most confident participants with regard to talking to and being understood by children and parents about pain were qualified nurses working in a clinical context, highlighting the importance of current clinical experience in relation to confidence to enact “pain talk.” The participants who felt less confident about “pain talk” were least experienced in “pain talk” and reported that additional training (eg, clinical assessment and management of pain, communication, and strategies such as distraction and play techniques) would be of value and were participants who were currently undergoing training.

### 3.3 Theme 1: contextualizing and assessing

Participants were aware that pain communication is a complex and multifaceted interpersonal exchange, and they tried to shape their “pain talk” to create a calming atmosphere for each child; this could only be achieved by gaining an understanding of the context of the situation. Knowledge of context included an awareness of child‐related characteristics such as the age of the child, developmental stage, verbal ability, level of understanding, and other factors (eg, physical problems such as hearing impairment, learning difficulties, and/or developmental disorders) that could impact on “pain talk.”

A wide range of additional factors also needed to be accounted for, such as the nature and possible cause of the pain, *“knowing how serious the situation is, how quickly decisions need to be made”* (P17), *“knowing whether the pain is acute or chronic”* (P45), child's pain history (including medication and other interventions), and any other *“underlying conditions*” (P112). Other factors such as how the child had managed any previous pain experiences and/or pain medication provided context with some participants reporting that *“children who 'hate' medication”* (P57) present additional challenges to creating effective “pain talk.” Issues such as the perceived *“willingness [of parents] to engage”* (P108) or the child being unhappy at school needed to be accounted for as these might be complicating the situation.

“Pain talk” was supported throughout by conscientious observation of the child’s and parent's non‐verbal cues, such as facial expressions, body tension, and distress, to generate as much contextual information as possible to help shape their communication and decision‐making. For example, participants described paying specific attention to examining *“parents’ body language”* (P8) and whether the parent *“is irate or upset”* (P22) when actively gathering contextual cues to inform “pain talk.”

### 3.4 Theme 2: empowering, explaining, and educating

“Pain talk” was reported as a means of empowering children and parents through providing them with information, explanations, and creating opportunities for education and, for some children and parents, opportunities for self‐management (eg, in managing postoperative pain at home). All of these factors aimed to ensure that the child/parents understood what was happening regarding pain assessment and management as this was seen to be a critical prerequisite for effective “pain talk.” However, understanding was influenced by the specific contextual factors such as the age of the child, culture, health literacy, and whether language barriers existed that reduced the fluency of conversation between the participants and the child/parents. In particular, language barriers were identified as being actually or potentially problematic regarding participants' ability to successfully engage with “pain talk” as *“interpreters are not always available”* (P26). Other factors reported as both shaping “pain talk” and influencing nurses' confidence included the *“parents’ education and literacy”* (P114).

The content and delivery of “pain talk” aimed for simplicity, transparency, and clarity; this involved using simple language *“in a way that the child could understand”* (P51). Participants concentrated on providing information that was concise, focused and repeated, if needed, to reinforce understanding about next steps and procedures. When talking about pain, the participants reported asking short‐ or open‐ended questions, avoiding technical jargon and medical terminology, using age‐appropriate language when talking directly to children, and deploying terms and language that were already used by children and parents. “Pain talk” aimed to involve a *“gentle*” (P141) approach, *“on [a] level with child in presence of parent”* (P70) to facilitate a collaborative inclusive conversation and subsequent pain management plan. A typical approach was to:

“Communicate to her [the child] clearly, informing her of everything but not using any medical jargon which she may find confusing or distressing” (P89).

Participants used “pain talk” to explain what to anticipate within consultations such as potential discomfort due to a dressing change or taste of a medication or potential side effects. “Pain talk” that explained the link between action and outcome was deemed important. For example, one participant described how they would clearly present this link between action and outcome by stating; *“I've brought you some medicine to take the pain away. It will make your throat feel better”* (P118). Providing information about the duration of the pain and associated discomfort was also considered to be important as explained by P95:

“I am going to change the dressing on your arm. Is that ok? It may hurt a little bit but we need to do it to keep it clean. I'll try to be as quick as I can. OK?” (P95).

“Pain talk” that provided information was reported to be a crucial element of explicit communication that aimed to make the child feel better. The purpose of “pain talk” was to offer reassurance and psychological comfort to children and parents. Through these processes, “pain talk” aimed to make a situation that was potentially distressing and unfamiliar to the child and parents as understandable and structured as possible, highlighting the importance of shared understanding about what procedures would happen and when.

Explanations and education provided by participants aimed to reduce parental misconceptions about pain and to help generate shared and realistic expectations regarding the outcome of interventions thus trying to avoid disagreements. Empowering children and parents through information and effective “pain talk” helped to address situations in which parents misinterpreted pain, for example, by overestimating, underestimating, denying, or mistaking their child's pain for temperamental manifestations. Specifically, one participant describes how parents may underestimate their child's pain due to their focus on the child's behavior.

“Parents may not feel confident to give pain relief and worry they are responding to poor behaviour rather than pain. I would suggest that often poor behaviour is the result of pain or discomfort and therefore pain relief will help” (P109).

Calm and reassuring “pain talk” was used to explain how analgesics worked, to children and parents trying to reduce *“possible frustration that analgesia can take time to work”* (P97) and explaining dosages and timing of medications such as emphasizing:

“….. the importance of regular analgesia post‐operatively (preventing pain rather than treating extreme pain) to gain cooperation” (P74).

Participants emphasized the importance of engaging with calming “pain talk” with parents, particularly in situations where parents *“want[ed] action and for the pain to be instantly taken away”* (P95) when this may not always be possible. Acknowledging differences in parental behavior and perceptions, participants described the need for adept and knowledgeable “pain talk” with parents reluctant for their child to have analgesia (eg, due to fears of opioid addiction).

Skillful “pain talk” was required to account for the parents’ emotional state (eg, anxious, stressed, fearful, guilty, agitated, distant) and to manage challenging situations where different perspectives on what was needed or achievable existed between participants, children, and parents. Participants reflected that the most complex conversations were ones where *“you have differing opinions of the nurse / family re. the pain or how it [pain] should be managed”* (P10). Such difficult situations where conflicting opinions existed between participants and parents regarding optimal pain management were exacerbated where previous clinical situations had resulted in parents losing trust in healthcare professionals regarding their child's care. This is illustrated by one participant who described the need for skillful “pain talk” that could accommodate:

“Angry, frustrated parents who have lost faith in HCP's [healthcare participants] to be able to manage their child's pain” (P23).

Verbal “pain talk” was supported through the use of written materials such as *“a pamphlet about the condition”* (P57) and through the use of other resources such as showing “*how [technique/care procedure] will be done on a doll”* (P35). Providing other modalities to support “pain talk” was deemed important to cement understanding around processes and provide access to readily available information after the conversation (eg, a pamphlet).

### 3.5 Theme 3: supporting, affirming, and confirming

“Pain talk” was key to supporting children and parents in the context of experiencing pain. Support was evident in different ways including providing reassurance, affirming and acknowledging the situation, and confirming that together they would manage the situation. Affirmation of the child in terms of praising the child verbally was a component of effective “pain talk” such as telling the child *“how brave they are”* (P47) as well as recognizing a child's endurance in a pain‐related situation.

Situating the child at the center of “pain talk” and acknowledging and validating their pain were key qualitative characteristics of pain communication:

“Acknowledging the situation…that she [child] is in pain and that I understand that and would like to help” (P69).

Offering reassurance was a component of “pain talk” with the emphasis on the promise that the child's pain would be appropriately examined with a view to finding the best possible solution:

“Give constant reassurance that it will get better and will feel less painful if she takes some medicine” (P66).

“Pain talk” was viewed as a way of supporting children by creating a space that would acknowledge the child's agency and allow them to enact their agentic status and exert some control over the situation. Support was confirming and evident in creating choices for children. Effective “pain talk” could create multiple, authentic child‐centered choices, for example, the form of medication *“liquid or tablets” (*P111), who to give the medication, *“mummy /daddy can help you take the meds [medication]”* (P68) or where to sit during the care procedure:

“Try to allow Sati to choose things like where she would like to sit for example to give her some control over the situation” (P23).

Participants acknowledged that the child's emotional state was fundamental to how they shaped their “pain talk” with the aim being to create support, rapport, familiarity, and some level of trust through sensitive and tailored communication:

“I take into account their age what they are likely to understand, and to ensure that they don't become frightened or anxious when talking about pain” (P29).

Engaging with effective “pain talk” was perceived as particularly important if the child reported negative previous experiences with care procedures and pain management. In such instances, participants sought to achieve effective “pain talk” through attending to prior experiences of pain, hospitalizations, and pain management and how patients and families had coped with these. Supportive “pain talk” aimed to reduce any distress and take account of *“memories of past experiences if they have not been positive ones”* (P09).

Affirmation of the child's experiences was an important component of “pain talk” generating a sense of trust and acknowledgment; *“gaining the trust from the family makes the conversation easier”* (P73). Affirmation was also evident in the ways in which participants reported asking parents to affirm their understanding of key elements of conversations, through techniques such as repetition of content and *“asking questions that suggest they are understanding and they can talk through the plan”* (P72).

### 3.6 Theme 4: protecting, distracting, and restoring

“Pain talk” aimed to protect children from the deleterious effects of pain by creating the best milieu for the individual child and their parent. Protective “pain talk” involved many factors such as “*ensuring you act in the best interests of all involved, not just what you think”* (P111) and using “*caring body language and effective listening to develop trust”* (P25). Protective “pain talk” aimed to ensure that each individual encounter was as positive as possible for the child and parent; this required a skillful titration of being open and not creating worry:

“how to communicate that a procedure may be painful without the parents becoming too anxious” (P91).

Participants reported that distraction was being a key component in their “pain talk” and that it could be *“a valuable analgesic”* (P110). Distraction could both promote communication and help protect children from focusing too much on the pain or procedure as, for example, using toys or teddies meant that the child could *“point to where teddy hurts as opposed to themselves”* (P39). *The* use of teddies in this manner also reflected the idea of developmentally appropriate medical play in terms of managing pain. Distraction took many forms such as *“using humour in a moderate way”* (P26) and *“age‐appropriate games* (P47). Distraction was tailored to the individual child; this tailoring was generated from what participants learned about the child through their “pain talk,” for example.

“I would try to engage Josh in light chat…finding out his likes hobbies etc trying to build a relationship with him. After a very careful history taking, I would examine Josh whilst still chatting and distracting him in order to determine the location of his pain” (P69).

Restoring was reported less frequently but “pain talk” was seen as a way of trying to create positive memories of the current episode or event and also recognizing that previous experiences may not have been positive. For many participants, the valence of children's previous medical encounters was critical as *“children's past experiences may cause anxiety and make things very difficult”* (P7). Consequently, participants' focused efforts on specifically asking about previous encounters to inform their restorative “pain talk” in the current situation, for example:

“If she said it hurt the last time we would talk about reasons and how we can make it better this time” (P26).

## 4 DISCUSSION

“Pain talk” can be defined as a triadic collaborative process of communication between nurses, children, and parents, requiring confidence and involving participants both talking and attending to children and parents about their pain and creating opportunities for the children and parents to engage in a reciprocal way. It was a welcome finding that the emphasis was placed on triadic communication rather than just dyadic (nurse‐to‐child, mother‐to‐child; ^5^, ^22^) or dyadic (nurse‐to‐parent) with no involvement of the child ^23^. Our study findings demonstrated multi‐fold aims of “pain talk” regarding improving communication about children's pain. Specifically, “pain talk” aimed to contextualize and assess and manage pain; to empower, explain, and educate; and to protect, distract, and restore. These core components were perceived by participants to be essential in ensuring that the agency of both the child and the parent was central to discussions and decisions made in relation to the child's experience of pain. Engagement with collaborative “pain talk” fostered reassurance and positive opportunities for children and parents and the potential for flourishing whereas routinized talk that was not tailored to the child, parent(s), and situation was perceived as something that could disempower the children and parents in terms of influencing the experience in any meaningful way. “Pain talk” facilitated the building of rapport between the nurse, child, and parent, a core “concept” in nursing work.^22^ However, “pain talk” did not just occur, and it required nurses who were confident, felt knowledgeable, and prepared to engage in “pain talk.”

Education and training were identified as critical components of good pain care and inherent in nurses' ability to effectively use “pain talk” to engage with children and parents. Improved education for nurses is a factor reported by other studies as being key to good pain communication; this pain education needs to be ongoing and woven into undergraduate and postgraduate nursing programs.^24^ There is an increasing evidence base that demonstrates the relationship between knowledge, confidence, and aspects of pain assessment and management. For example, clinician education leading to increased knowledge is shown to improve confidence in using the FLACC Pain Scale.^25^ Additionally, a multifaceted knowledge translation intervention designed to improve the vaccination experience in schools resulted in nurses reporting both increased confidence in their ability to assess child pain and fear and enhanced collaboration among staff and students.^26^ Moreover, Simons' work highlighted the importance of nurses' confidence alongside good leadership and adequate resources in terms of being critical components of effective pain management.^27^ True partnership, collaboration with, and empowerment of parents require nurses to be knowledgeable and confident.^28^

This sense of confidence was critical to the success of nurses’ delivery and engagement with “pain talk.” Broadly, nurses were using “pain talk” to engage with children and parents to minimize potential distress and adverse effects. This aligns with Wong's concept of atraumatic care as care given by healthcare personnel using interventions that eliminate or minimize distress (both psychological and physical) experienced by children and their families.^29^ Despite not yet being fully embedded in clinical pediatric practice, alignment to the concept of atraumatic care was evident in the nurses’ “pain talk” and their understanding of the importance of minimizing distress. More contemporary approaches to atraumatic care can be seen in the move to the use of psychologically informed care which aims to mitigate the challenges that procedures and interventions pose to children's short‐ and longer‐term mental health and well‐being.^30^ Improvements such as promoting a soothing presence, promoting feelings of safety, providing appropriate emotional support, developing a healing environment, empowering children, and informing therapeutic practice^30^ are evident in both nurses' “pain talk” and the efforts the nurses in our study went to create a positive “pain” experience for the children. Our findings in relation to nurses using protective and restoring “pain talk” are congruent with the concept of psychologically informed health care and efforts to align with existing evidence for effective pain management that supports the need to empower and support children and promote feelings of safety and help children create good memories^9^ of procedures or painful experiences. “Pain talk” involved comfort and reassurance that a good solution would be identified and that children would be able to have choice and control and be agentic, as seen in nurses' emotion work relating to pain in a pediatric oncology clinic.^31^ Additionally, study findings showed that nurses embraced the concept of trauma‐informed care by asking children about any previous negative pain and wider medical experiences, using this knowledge to inform their attempts to better assess and manage children's pain.^32^

While our study findings reassuringly highlighted the incredible efforts that nurses reported they adopted to engage in effective “pain talk,” it is important to consider how this “pain talk” and nurses' actions are perceived by others, namely children and their parents. Additionally, it is important to consider how such interactions occur in real‐life settings. Vasey and colleagues' qualitative work interestingly identified that while nurses reported wishing to involve parents as partners in management of their child's pain, nurses did not consistently involve parents in their efforts to manage children's pain.^28^ Such findings highlight the importance of studying the wider social context of pain assessment and management to evaluate the success of attempts to manage children's pain.

Our study findings highlighted the importance of nurses generating multifactorial information regarding the specific context of the pain situation to enable them to shape effective “pain talk” and provide optimal pain management for children. To achieve this, nurses in this study noted the importance of addressing health literacy. While the pain literature has typically addressed health literacy from an adult perspective,^33^, ^34^ it is important to consider both child and parental health literacy when discussing child pain. Numerous factors have been shown to influence children's health literacy such as the child's agency, the role of other individuals (eg, parents) in addition to disease and other health‐related factors.^35^ Importantly, nurses in this study also highlighted the importance of parental health literacy, describing how levels of parental health literacy were deemed to be important in terms of “shaping” how they delivered “pain talk,” the degree to which they felt confident to engage with “pain talk”; and their confidence around how this “pain talk” was understood by both children and parents. Despite a dearth of literature concerning parental health literacy in the context of pediatric pain, findings from the broader pediatric literature have demonstrated low levels of parental health literacy to be associated with child health behaviors that may impact child health and well‐being,^36^ child outcomes,^37^ and higher levels of parental anxiety.^38^ The lack of specific research around child and parental health literacy and pediatric pain identifies an important area for future research given the commonality of the experience of pain in childhood.

Strengths of this study include the size of the participant sample and the international nature of the sample, resulting in responses from participants across a range of 11 countries in the Northern and Western Hemispheres. Such an international representation of findings provides important information regarding the nature of “pain talk” across different settings. However, the sample described in this study lacks diversity regarding gender, ethnicity, and socioeconomic status of countries. Notably, only 6 of the 141 nurses (4.3%) were men, 92.9% of the sample reported their ethnicity to be White and responses were collected from well‐resourced countries. Future research in this area should helpfully sample a wider range of men (male nurses), a more diverse sample of nurses in terms of ethnicity and socioeconomic characteristics of resident countries. Ensuring diversity across as broad range of factors as possible is important to understand the complexities regarding how nurses engage in “pain talk” in varied situations, particularly given the focus of nurses on shaping “pain talk” according to the specific context of the child's situation.

In conclusion, our study reveals that “pain talk” has multiple aims; it aims to improve communication about children's pain and minimize potential pain‐related distress and adverse effects. Findings showed that the nature of “pain talk” was varied, involving provision of information, support, comfort, and reassurance. For nurses in this study, effective “pain talk” aimed to promote the agency of the child and parent and their engagement in discussions and decision‐making. Importantly, nurses adapted their “pain talk” to the context of the child's pain, previous experiences, and current concerns to promote optimal pain management. Overall, study findings show how “pain talk” can be used by nurses across a range of settings to improve the management of children's pain.

## CONFLICT OF INTEREST

The authors confirm that they have no conflicts of interest to declare.

## Supporting information

Appendix S1

Appendix S2
