# Supplementary material for: “Pain talk”: A triadic collaboration in which nurses promote opportunities for engaging children and their parents about managing children’s pain
Source: Paediatr Neonatal Pain. 2021 Aug 9;3(3):123–33. doi: 10.1002/pne2.12061 (PMC8975224; doi:10.1002/pne2.12061)
Supplement: Supplementary file 1 — Appendix S1 [file PNE2-3-123-s002.docx]

**Appendix 1: Survey content including vignettes**

| **Domain 1: Participant information** | | |  |
| --- | --- | --- | --- |
| A. Demographic information | | (1) Gender  (2) Ethnicity  (3) Country of residence | Closed  Closed  Closed |
| B. Work-related information | | (1) Where did you undertake your nursing training?  (2) What is your job title? (e.g., Staff Nurse, Ward Nurse, Ward Manager, Professor, Director of Nursing)  (3) How many years since you first qualified as a nurse?  (4) What is your qualification status and setting?  (5) I have a children’s nursing/child health qualification (YES/NO)  (6) What particular setting do you work in?  (7) Which children age groups do you work with?  (8) When did you most recently receive education/training in managing children’s pain?  (9) Can you tell us about any particular training that you have received in managing children’s pain? | Closed  Open-ended  Closed  Closed  Closed  Closed  Closed  Closed  Open-ended |
| **Domain 2: General questions about pain communication** | | |  |
| # | Exact question | | Type of question |
| 1 | Typically, what factors influence the language you use and the way you talk to children and their parents? | | Open-ended |
| 2 | How confident do you feel when talking to a CHILD who is experiencing pain? | | Closed  (1 = not at all confident, 5 = Very confident) |
| 3 | How confident do you feel when talking to a PARENT whose child is experiencing pain? | | Closed  (1 = not at all confident, 5 = Very confident) |
| 4 | What factors might influence how confident you feel about talking to a child and/or their parent about pain? | | Open-ended |
| 5 | How confident do you typically feel that a CHILD has understood what you have said about managing their pain? | | Closed  (1 = not at all confident, 5 = Very confident) |
| 6 | How confident do you typically feel that a PARENT has understood what you have said about managing their child’s pain? | | Closed  (1 = not at all confident, 5 = Very confident) |
| 7 | How do you know whether a child and/or parent has understood what you have said about managing the child’s pain? | | Open-ended |
| 8 | What factors might influence how confident you feel concerning the extent to which the child and/or parent has understood what you have told them about the child's pain? | | Open-ended |
| 9 | What are the biggest challenges when talking to children and/or parents about managing children’s pain? | | Open-ended |
| **Domain 3: Vignettes** | | | |
| Josh | Josh is 9 years old and was admitted to hospital via A&E having developed “really bad pain” in his tummy at school. You meet Josh and his mum on the surgical ward. | | |
| Sati | Sati is 3 years old and is attending the hospital with her Dad. She is about to have a dressing on her arm changed. This procedure could be painful. | | |
| Mikel | Mikel is 4 years old and you want to give him some ibuprofen for his pain following a tonsillectomy. | | |
| Lisa | Lisa is 10 years old and you want to give her some paracetamol for her pain. She has a fractured wrist which has just been put into a splint. | | |
| **Domain 4: Questions about pain communication concerning each vignette** | | | |
| # | Question wording | | Type of question |
| 1 | What would you say in this particular situation? | | Open |
| 2 | How you would approach this particular situation? E.g., are there particular techniques that you might use to support the way that you communicate with children like Josh and/or their parents? If so, what are these and why would you use them? | | Open |
| 3 | Would anything enhance your communication with children and/or parents in this particular situation? (e.g., particular strategies, training). | | Open |
| 4 | How confident would you feel in your own practice providing care to a child in this particular situation? | | Open |
| 5 | How often have you treated a child in this particular situation in your own practice? | | Open |
| 6 | Any other comments (e.g., key challenges)? | | Open |
