# Supplementary material for: “Pain talk”: A triadic collaboration in which nurses promote opportunities for engaging children and their parents about managing children’s pain
Source: Paediatr Neonatal Pain. 2021 Aug 9;3(3):123–33. doi: 10.1002/pne2.12061 (PMC8975224; doi:10.1002/pne2.12061)
Supplement: Supplementary file 2 — Appendix S2 [file PNE2-3-123-s001.docx]

**Appendix 2: Confidence ratings regarding pain communication with children and parents according to participant qualification status**

| Qualification status | Mean confidence when talking to a child experiencing pain (SD) | Mean confidence when talking to a parent whose child is experiencing pain (SD) | | Mean confidence regarding degree to which child has understood what you have said about managing their pain (SD) | Mean confidence regarding degree to which parent has understood what you have said about managing their child’s pain (SD) |
| --- | --- | --- | --- | --- | --- |
| Pre-registration student nurse currently undertaking a course to gain a children’s nursing qualification. | 3.36 (0.63) | 3.13 (0.80) | | 3.00 (0.61) | 3.41 (0.79) |
| Qualified nurse currently undertaking a course to gain a children’s nursing qualification. | 3.75 (0.50) | 3.75 (0.50) | | 3.50 (1.29) | 3.75 (0.96) |
| Qualified nurse currently working in a clinical capacity | 4.41 (0.72) | 4.42 (0.64) | | 3.71 (0.76) | 4.04 (0.66) |
| Qualified nurse currently working in academia. | 4.15 (0.80) | 4.00 (1.22) | | 3.62 (0.87) | 3.77 (0.73) |
| Qualified nurse currently working in another setting (e.g. school). | 4.14 (0.38) | 4.00 (0.58) | | 3.43 (0.79) | 4.00 (1.00) |
|  | | |  |  |  |
|  | | |  |  |  |
|  | | |  |  |  |

† Data provided from 137 participants as 4 missing responses across all cells.

‡ All responses relate to 1-5 numerical rating scale with 1= not at all confident and 5=very confident.
